# Taxonomic revision of the genus *Glochidion* (Phyllanthaceae) in Taiwan, China

**DOI:** 10.3897/phytokeys.159.54839

**Published:** 2020-09-03

**Authors:** Gang Yao, Zhu-Qiu Song, Bin-E Xue, Shi Shi, Yu-Ling Li, Shi-Xiao Luo

**Affiliations:** 1 College of Forestry and Landscape Architecture, South China Agricultural University, Guangzhou 510642, China South China Botanical Garden, Chinese Academy of Sciences Guangzhou China; 2 Key Laboratory of Plant Resources Conservation and Sustainable Utilization, South China Botanical Garden, Chinese Academy of Sciences, Guangzhou 516650, China Zhongkai University of Agriculture and Engineering Guangzhou China; 3 College of Horticulture and Landscape Architecture, Zhongkai University of Agriculture and Engineering, Guangzhou 510225, Guangdong, China South China Agricultural University Guangzhou China

**Keywords:** lectotypification, new species, new synonym, Phyllantheae, taxonomy

## Abstract

A comprehensive taxonomic revision of the genus *Glochidion* J.R. Forst. & G. Forst. from Taiwan in China was carried out based on the examination of herbarium specimens and filed investigations. Eight species and three varieties are recognized, including a new species endemic to Taiwan, *G. lanyuense* Gang Yao & S.X. Luo, which is described and illustrated. Three names, viz. *G. chademenosocarpum* Hayata, *G. kusukusense* Hayata, and *G. ovalifolium* F.Y. Lu & Y.S. Hsu, are reduced to the new synonyms of *G. rubrum* Blume, *G. lanceolatum* Hayata, and *G. ellipticum* Wight, respectively. Two names, viz. *G. lanceolatum* Hayata and *G. suishaense* Hayata, are lectotypified here. A key to the *Glochidion* species in Taiwan is provided.

## Introduction

*Glochidion* J.R. Forst. & G. Forst. is the second largest genus within the tribe Phyllantheae Dumort. (Phyllanthaceae Martinov) (Govaerts et al. 2000; Webster 2014; Duocet Group 2016 onwards). It is represented by more than 300 species of shrubs or trees distributed primarily in the Indo-Pacific, east to southeast Polynesia and south into Australia (Govaerts et al. 2000; Li and Gilbert 2008), with about 30 species in China (Li and Gilbert 2008; Yao and Zhang 2015a; Yao et al. 2017). Molecular phylogenetic studies have shown that *Glochidion* and some other genera (viz. *Breynia* J.R. Forst. & G. Forst., *Phyllanthodendron* Hemsl. and *Sauropus* Blume) were nested deeply within the large and morphologically heterogeneous genus *Phyllanthus* L. s.s. (over 800 species) (Hoffmann et al. 2006; Kathriarachchi et al. 2006; Pruesapan et al. 2012; van Welzen et al. 2014). Therefore some authors suggested the inclusion of these genera in *Phyllanthus*, and accepted the concept of *Phyllanthus* s.l. (over 1200 species; including *Breynia*, *Glochidion*, *Phyllanthodendron* and *Sauropus*) (Hoffmann et al. 2006; Wagner and Lorence 2011). However, others suggested that it might be more reasonable to disintegrate *Phyllanthus* s.s. into smaller genera, and accept the generic status of *Glochidion* and other relevant genera (Pruesapan et al. 2012), which is further supported in morphological (van Welzen et al. 2014), palynological (Yao and Zhang 2016) and wood anatomical (Jangid and Gupta 2016) analyses. Thus, the generic name *Glochidion* is still accepted widely in recent taxonomic literature (e.g. van Welzen et al. 2014; Webster 2014; Ramana et al. 2015; Chia et al. 2017; Yao et al. 2017; Chakrabarty and Balakrishnan 2018; Xu et al. 2020).

Taxonomic studies of *Glochidion* have largely been conducted at the regional level, such as in China (Li 1994; Li and Gilbert 2008), Indo-Burma (Chakrabarty and Balakrishnan 2018), Indo-China (Beille 1927), Indian subcontinent (Chakrabarty and Gangopadhyay 1995), Java (Backer and Bakhuizen 1963), the Philippines (Robinson 1909), Sumatra (Airy Shaw 1981), Thailand (Airy Shaw 1972; van Welzen 2007) and Vietnam (Nguyen 2007). Thus, a comprehensive taxonomic revision of the genus is still lacking, and an acceptable infrageneric classification system of the genus has not been proposed. In *Flora Republica Popularis Sinicae*, the Chinese *Glochidion* species were classified into two sections based on the number of stamens, viz. sect. Glochidiopsis (Blume) Hook.f. (stamen 3) and sect. Multandrum P.T. Li (stamen 4–8) (Li 1994), but this classification system was not supported in molecular phylogenetic studies (Kawakita et al. 2004; Luo et al. 2017). In China, the taxonomic study of *Glochidion* in Taiwan has a long history. Forbes and Hemsley (1894) were the first authors to report *Glochidion* species from Taiwan and three species were reported then, viz. *G. arnottianum* Müell. Arg., *G. fortunei* Hance, and *G. hongkongense* Müell. Arg. Subsequently, a number of taxonomic studies of Taiwanese *Glochidion* were conducted (e.g., Hayata 1903, 1904, 1920; Kanehira 1936; Croizat and Hara 1940; Keng 1955; Li 1963; Hsieh 1977; Deng and Wang 1993; Li 1994; Hsu et al. 2006; Li and Gilbert 2008). In the latest taxonomic monograph accomplished by Hsu et al. (2006), they reviewed the taxonomic history of Taiwanese *Glochidion* and recognized nine species, viz. *G. acuminatum* Müell. Arg., *G. hirsutum* (Roxb.) Voigt, *G. kusukusense* Hayata, *G. lanceolatum* Hayata, *G. ovalifolium* F.Y. Lu & Y.S. Hsu, *G. philippicum* (Cavan.) C.B. Rob., *G. puber* (L.) Hutch., *G. rubrum* Blume, *G. zeynanicum* (Gaertn.) A. Juss. Among some of these studies, four taxa described from Taiwan [viz. G. assamicum (Müll. Arg.) Hook. f. var. magnicapsulum Croiz. & Hara., *G. chademenosocarpum* Hayata, *G. kusukusense*, and *G. suishaense* Hayata] have long been treated as dubious taxa because relevant specimens, especially the types, were unavailable (Kanehira 1936; Keng 1955; Deng 1992; Deng and Wang 1993; Hsu et al. 2006). Although the latter three species were accepted in *Flora Reipublicae Popularis Sinicae* (Li 1994) and *Flora of China* (Li and Gilbert 2008), their morphological descriptions were merely derived from their protologues without further observations. Hsu et al. (2006) accepted the species *G. kusukusense* and synonymized the name *G. suishaense* under *G. rubrum*, but types of the two species were still not referred to in their study.

**Figure 1.**
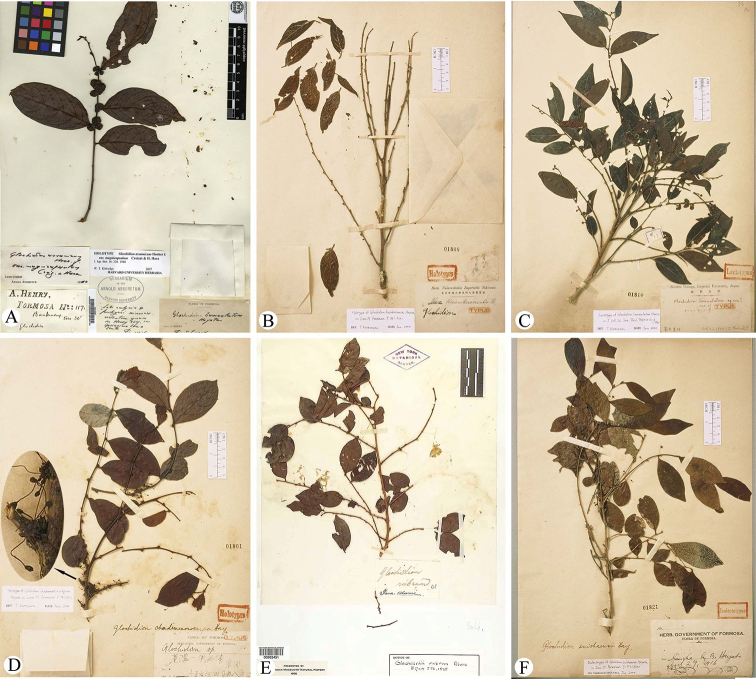
**A** holotype of Glochidion assamicum var. magnicapsulum Croiatz & Hara (*A. Henry 117*, A) **B** holotype of *G. kusukusense* Hayata (*B. Hayata & S. Sasaki s.n.*, TI) **C** lectotype of *G. lanceolatum* Hayata (*T. Makino s.n.*, TI) **D** holotype of *G. chademenosocarpum* Hayata (*B. Hayata s.n.*, TI) **E** isotype of *G. rubrum* Blume (*C.L. von Blume s.n.*, NY) **F** isolectotype of *G. suishaense* Hayata (*B. Hayata s.n.*, TI).

In our taxonomic study of the genus *Glochidion*, types of aforementioned enigmatic taxa were found in the herbaria A (G. assamicum var. magnicapsulum, Fig. 1A) and TI (*G. chademenosocarpum*, Fig. 1D; *G. kusukusense*, Fig. 1B; *G. suishaense*, Fig. 1F), and a collection of the genus from Lanyu island of Taiwan, China, was found to be very different from all of the other *Glochidion* species recorded from Taiwan and adjacent regions. Thus, a comprehensive taxonomic revision of *Glochidion* in Taiwan was conducted in this study.

## Material and methods

Specimens of *Glochidion* deposited in the herbaria A, HAST, K, KUN, IBSC, LINN, MA, NAS, NCAI, NY, P, PE, PH, TAI and TI, were studied carefully in the present study. Field investigations of Taiwanese Phyllantheae species were also conducted from 2015 to 2019. Additionally, most materials of Taiwanese *Glochidion*, which were obtained by Dr. A. Kawakita from Kyoto University, Japan, in his recent field studies of the co-evolutionary system involving *Glochidion* plants and *Epicephala* moths (Kawakita et al. 2004; Kato and Kawakita 2017), were generously provided for the present study. Morphology of leaves, styles and capsules, as well as the number of ovaries and stamens, were all studied carefully. Herbarium abbreviations cited here are based on the Index Herbarium of Thiers (2013 continuously updated).

## Results

In total, over 800 specimens were examined in the present study. Morphological studies based on the careful examination of herbarium specimens and extensive filed investigations revealed that eight species and three varieties of *Glochidion* should be recognized in Taiwan, China, viz. G. acuminatum var. acuminatum, *G. ellipticum* Wight, *G. lanceolatum*, *G. lanyuense* Gang Yao & S.X. Luo, *G. philippicum*, *G. puber*, *G. rubrum*, G. zeylanicum var. zeylanicum, G. zeylanicum var. tomentosum Trim., among which the species *G. lanyuense* is new to science. Additionally, the three names *G. chademenosocarpum*, *G. kusukusense* and *G. ovalifolium* should be reduced to the new synonyms of *G. rubrum*, *G. lanceolatum* and *G. ellipticum*, respectively. Because other species were morphologically described in detail by previous authors (e.g. Hsieh 1977; Deng and Wang 1993; Hsu et al. 2006; Li and Gilbert 2008), we only provide a morphological description for the new species *G. lanyuense*, but a key to all of the *Glochidion* species in Taiwan is presented.

## Taxonomic treatment

### 1. Glochidion acuminatum

Taxon classificationPlantaeMalpighialesPhyllanthaceae

Müll. Arg., Linnaea 32: 68. 1863

366CE058-B5DB-566F-B266-ED97CA222249

Figure 2A–C

Bradleia acuminata Wallich, Numer. List 7855. 1847, nom. nud. Basionym.

#### Type.

Nepal. “Nepalia”, *Wallich 7885* (lectotype: K-000246416, photo!, designated by Yao and Zhang 2015b).

**Figure 2.**
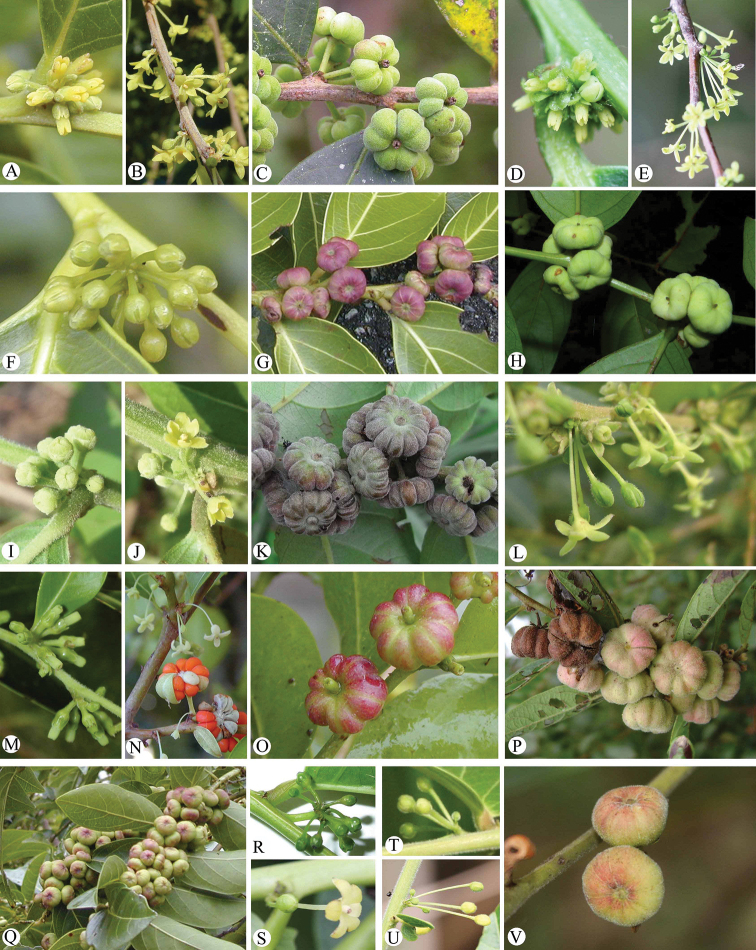
General morphology of *Glochidion***A–C**G. acuminatum var. acuminatum Müll. Arg **D, E, H***G. ellipticum* Wight **F, G***G. lanceolatum* Hayata **I–K***G. philippicum* (Cav.) C.B. Rob **L, P***G. puberum* (L.) Hutch **M–O***G. rubrum* Blume **Q–S**G. zeylanicum var. zeylanicum (Gaertn.) A. Juss **T–V**G. zeylanicum var. tomentosum Trimen **A, D, F, I, M, R, T** female flowers **B, E, J, S, U** male flowers **C, G, H, K, N–Q, V** fruits **L** female flowers and male flowers **N** male flowers and fruits. Photographs: **A–C, F, G, I–K, M–O, Q–U** by A. Kawakita (Kyoto University, Japan) **D–E, H, L, P** by G. Yao **V** Z.Q. Song.

### var. acuminatum

Taxon classificationPlantaeMalpighialesPhyllanthaceae

872268C1-7D4F-5CC5-856F-36310AE0D528

Figure 2A–C

Glochidion hayatae Croiz. & Hara, in J. Jap. Bot. 16: 316. 1940. Type: China. Formosa (now Taiwan). Holisha, Giochi. 28 April 1916, *B. Hayata s.n.* (holotype: TI-01804, photo!).

#### Distribution and habitat.

The typical variety G. acuminatum var. acuminatum is widely distributed from India, Nepal, through Indo-China Peninsula, to China and Japan. In China, it occurs widely from the southwestern area to Taiwan. It grows in evergreen broad-leaved forests, open forests, valleys, or near streams, usually from low altitude to 2600 m. The species is recorded from Ilan and Taipei Hsien, through Nantou and Taichung Hsien, to Kaosiung and Pingtung Hsien, in Taiwan.

#### Taxonomic discussion.

Two taxa are described under the species *G. acuminatum*, the typical variety G. acuminatum var. acuminatum and the variety G. acuminatum var. siamense Airy Shaw. The species is represented in Taiwan by the typical variety, and another variety is distributed in Thailand and Yunnan province of China (Li and Gilbert 2008; Yao and Zhang 2015b). A detailed morphological comparison between the two taxa can be referenced in Yao and Zhang (2015b). The typical variety can be distinguished from all other Taiwanese *Glochidion* species by its small (5–7 mm in diameter) and deeply 6- or 8-grooved capsules (Figure 2C).

#### Representative specimens examined.

China. Taiwan. Kaosiung Hsien, Shanping Station, at an elevation of 1000 m, 7 November 1991, *C.C. Wang 818* (HAST); Ilan Hsien, Fushan, at roadside, 26 April 1992, *S.L. Chen 927* (HAST); Nantou Hsien, at an elevation of 650 m, 5 October 2001, *C.M. Wang 04509* (IBSC, PE); Nantou Hsien, Yuchih Hsiang, Lienhuachih, 23°55'08"N, 120°52'41"E, at an elevation of 640 m, 24 April 1996, *C.N. Chen et al. 03316* (KUN); Nantou Hsien, Lienhuachi, 23°55'17"N, 120°54'20"E, 6 July 1936, *K. Mori 1527* (TAI); Nantou Hsien, Lienhuachi, 23°53'53"N, 120°52'58"E, 24 July 1955, *Y. Keng & Liu et al. s.n.* (TAI); Nantou Hsien, Jenai Hsiang, Hui-Sun Experimental Forest, 24°05'34"N, 121°01'27"E, at an elevation of 660 m, 5 October 2000, *C.M. Wang 04509* (IBSC, PE); Pingtung Hsien, Kueitsuchia, 21°57'56"N, 120°48'18"E, 1 January 1917, *E. Matuda 1177* (TAI); Taichung Hsien, forest margin, at an elevation of 900 m, 18 April 2003, *C.M. Wang 6609* (HAST); Taipei Hsien, Chutsuhu, 25°10'9"N, 121°31'54"E, 16 November 1969, *C.C. Hsu 6561* (TAI); Taipei Hsien, Neihu, Naihosyo, 25°4'32"N, 121°34'49"E, 21 July 1973, *C.M. Kuo 3640* (TAI); Taipei Hsien, Neihu, 25°05'0"N, 121°34'0"E, 16 October 1993, *S.Y. Lu 24147* (PE); Taipei Hsien, Sekitei, 24°59'21"N, 121°38'57"E, 6 July 1949, *K. Kao 1350* (TAI); Taipei Hsien, Wulai, Urai, 24°51'47"N, 121°32'34"E, 25 October 1929, *S. Suzuki 3268* (TAI).

### 2. Glochidion ellipticum

Taxon classificationPlantaeMalpighialesPhyllanthaceae

Wight in Icon. Pl. Orient. 5: t. 1906. 1852

8419B28E-8278-599E-8140-9E675C46B021

**Figure 3.**
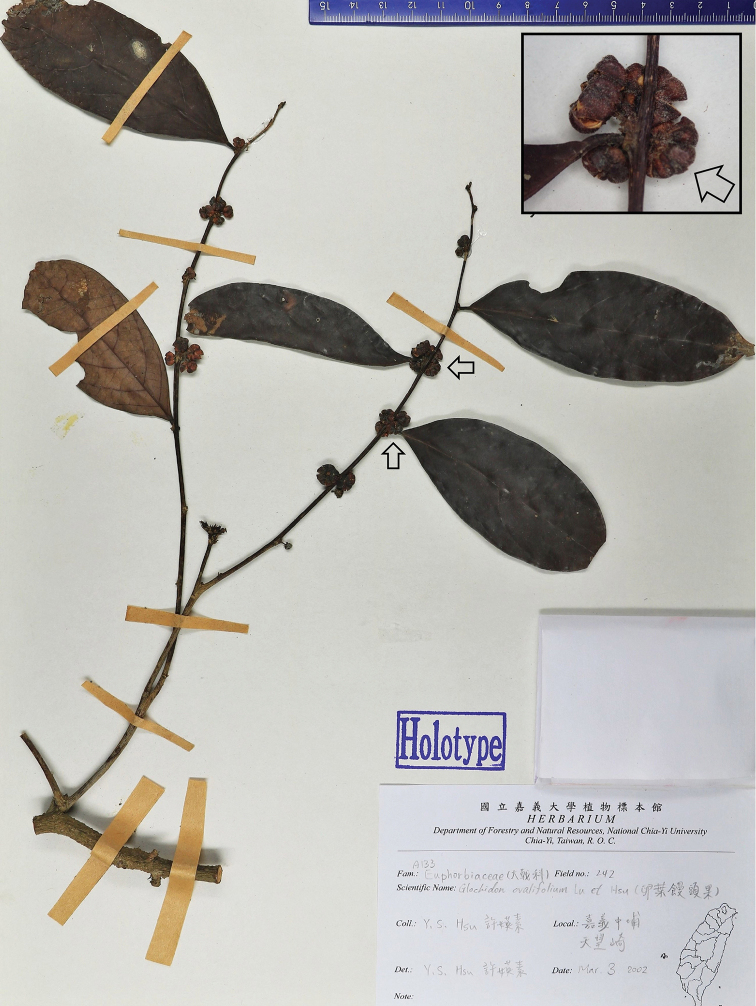
Holotype of *Glochidion ovalifolium* F.Y. Lu & Y.S. Hsu. (*F.Y. Lu* & *Y.S. Hsu 242*, NCAI!).

Figures 1A , 2D, E, H , 3

Phyllanthus assamicus Müll. Arg. in Flora 48: 378. 1865. Glochidion assamicum (Müll.-Arg.) Hook. f. in Fl. Brit. India 5(14): 319. 1887. Type: India, upper Assam, 1861, *J.D. Hooker* & *T. Thomson s.n.* [Glochidion 51] (lectotype: G -00324994, designated by Chakrabarty and Balakrishnan 2018); Remaining syntypes: India, Sikkim, 100 ft, 1861, *J.D. Hooker s.n.* [Bradleia 45] (CAL, herb. acc, no. 403548; G-00324992; NY-00263421); India, Assam, *Jenkins 530* (CAL).
Glochidion assamicum var. magnicapsulum Croiatz & Hara, in J. Jap. Bot. 16: 319. 1940. Type: China. Formosa (now Taiwan), September 1938, *A. Henry 117* (holotype: A!; isotype: NY) (Figure 1A).
Glochidion ovalifolium F.Y. Lu & Y.S. Hsu, in Quarterly J. For. Res. 25(4): 87. 2003. syn. nov. Type: China. Taiwan: Chiayi Hsien, Chungpu, 3 March 2002, *F.Y. Lu* & *Y.S. Hsu 242* (holotype: NCAI!, Figure 3; isotype: NCAI!).

#### Type.

India, Malabar, *R. Wight* Kew Distrib. No. 2663 (lectotype: K-000246408, photo!, designated by Chakrabarty and Balakrishnan 2018; isolectotypes: CAL; L-0030051, photo!); Remaining syntype: India, Malabar, *R. Wight 2576* (K!, K00024606; S!, S08-1933).

#### Distribution and habitat.

The species is widely distributed from northeastern India, Nepal, through Indo-China Peninsula, to China. In China, it occurs widely from the southwestern area to Taiwan. It usually occurs in evergreen broad-leaved forests, scrub on stream banks, roadsides, usually from low altitude to 1800 m. In Taiwan, the species is widely distributed from Keelung and Taoyuan, to Chiayi, Kaosiung, Nantou, Pingtung, Taichung and Tainan.

#### Taxonomic discussion.

Croizat and Hara (1940) described the variety G. assamicum var. magnicapsulum from Taiwan, and considered that it differed from the typical variety G. assamicum var. assamicum by its large female flowers (ca. 2.5 mm in diameter) and the pubescent and large capsules (8–10 mm in diameter). However, this variety had long been treated as a dubious taxa, or even not referred to in latter taxonomic treatments since its publication (e.g. Keng 1955; Hsieh 1977; Deng and Wang 1993; Hsu et al. 2006), until it was reduced to be a synonym of *G. ellipticum* (*G. assamicum* was cited as one of its synonyms) in *Flora of China* (Li and Gilbert 2008). After observing the types and many non-type specimens of *G. ellipticum*, it was found that the species showed much variation in morphology, such as its leaves are elliptic, lanceolate, oblong or ovoid in shapes; ovary usually 3–4-locular, and rarely 5-locular; fruits sub-glabrous or sparsely pubescent, usually 6–8 mm in diameter, and sometimes could be up to 10 mm in diameter. So the treatment of Li and Gilbert (2008) is accepted and the distribution of *G. ellipticum* in Taiwan is confirmed here. More Taiwanese specimens of *G. ellipticum* were also found and examined in the present study.

*Glochidion ovalifolium* was described from Chiayi, Taiwan, China, and it was suggested to be similar to *G. lanceolatum* in morphology, but differs in having hairy ovaries and fruits (Lu and Hsu 2003). However, results from checking the types of *G. ovalifolium* (Figure 3) revealed that the species is actually conspecific with *G. ellipticum* (Figure 1A), which is very different from *G. lanceolatum* by its female flowers and fruits cluster in axillary (vs. usually pedunculated supra-axillary cymes), ovary and fruits sub-glabrous or sparsely pubescent (vs. glabrous), stamens 3 (vs. 4–6). The pedunculated supra-axillary cymes of *G. ovalifolium* as described in its protologue, and observed in its line drawing provided in Lu and Hsu (2003), are incongruous with its types (Figure 3). Hence, based on a careful study of its type and non-type specimens, *G. ovalifolium* is here reduced to a new synonym of *G. ellipticum*.

#### Representative specimens examined.

China. Taiwan. Chiayi Hsien, Meishan Hsiang, Juifeng Bridge-Juili, 23°33'N, 120°39'E, at an elevation of 600 m, 25 August 2001, *C.M. Wang 05251* (IBSC); Zhuqi Hsiang, 5 October 2014, *H.Y. Chen 011* (NCAI); Kaosiung Hsien, Maoning Hsiang, at an elevation of 800 m, 27 January 1989, *J.C. Wang 5177* (HAST); Kaosiung Hsien, Liouhguei, roadside, at an elevation of 250–350 m, 5 May 1991, *M.J. Deng & S.L. Chen 463* (HAST); Keelung Hsien, Denryoko, 25°8'2"N, 121°44'26"E, May 1931, *Y. Yamamoto s.n.* (TAI); Nantou Hsien, Chingshuikou, 23°47'16"N, 120°46'55"E, 16 February 1959, *K.K. Huang 839* (PH); Nantou Hsien, Lienhuachih-Yuchih, 24 July 1955, *H. Keng* & *K. Liu s.n.* (PH); Nantou Hsien, 11 May 1991, *Y.S. Hsu* & *J.C. Liaw 198*, *199*, *201*, *202*, *203*, *204*, *207*, *208*, *209* (NCAI); Pingtung Hsien, at an elevation of 1427 m, 8 February 2015, *Y.J. Lin 021* (NCAI); Taichung Hsien, 25 July 1955, *H. Keng*, *Liu & Kao s.n.* (PH); Tainan Hsien, Kuantzuling, 23°20'32"N, 120°29'33"E, 3 May 1943, *Senben 390* (TAI); Taoyuan, 9 September 1990, *Y.S. Hsu* & *J.C. Liaw 70* & *71* (NCAI).

### 3. Glochidion lanceolatum

Taxon classificationPlantaeMalpighialesPhyllanthaceae

Hayata in J. Coll. Sci. Univ. Tokyo 20: 16. 1904

DB527E01-9340-582F-9FF1-EE1ABA0F769A

Figures 1B, C , 2F, G , 5A, C, E, G, I, L

Glochidion kotoense Hayata in Icon. Pl. Form. 9: 96. 1920. Type: China. Formosa (now Taiwan), Kôtôshô, *Anonymous s.n.* (holotype: TI-01807, photo!).
Glochidion sphaerostigmum Hayata in Icon. Pl. Form. 9: 96. 1920. Type: China. Formosa (now Taiwan), Suisha, *Anonymous s.n.* (holotype: TI-01817, photo!).
Glochidion kusukusense Hayata in Icon. Pl. Formos. 9: 96. 1920. syn. nov. Type: China. Formosa (now Taiwan), Kusukusu, July 1912, *B. Hayata* & *S. Sasaki s.n.* (holotype: TI-01808, photo!, Figure 1B).

#### Type.

China. Formosa (now Taiwan), Kelung, 31 October 1896, *T. Makino s.n.* (lectotype: TI-01810, photo!, Figure 1C; here designated); Remaining syntype: Taiwan, Kelung, 1 November 1896, *C. Owatari s.n.* (TI-01811, photo!).

#### Distribution and habitat.

*Glochidion lanceolatum* is distributed in China (only in Taiwan) and south Japan, and also recorded from the Philippines (Govaerts et al. 2019). It usually occurs in open forests, roadsides, and at low altitudes. The species is widely distributed from northern to southern Taiwan.

#### Taxonomic discussion.

The morphological description of *G. kusukusense* provided by Hayata (1920) is brief and short, and it is in accordance with its type that has only a short branch and several leaves (Figure 1B). In the protologue, *G. kusukusense* was compared morphologically with *G. wrightii* Benth, a species widely distributed in southern and southwestern China. After observing the type of *G. kusukusense* deposited in herbarium TI (Figure 1B), we found that its glabrous habit and lanceolate leaves were identical to that of the species *G. lanceolatum* (Figure 1C), which is widely distributed in Taiwan. Thus, we considered that *G. kusukusense* is conspecific with *G. lanceolatum* (Figure 1C) and reduced it to be a new synonym of the latter.

In Hsu et al. (2006) study, some specimens collected from Nantou and Taoyuan of Taiwan (out of the type locality of *G. kusukusense*) cannot be identified as *Glochidion* species usually known to Taiwan, while their lanceolate leaves and glabrous pedicel of male flowers observed seemed to be consistent with the diagnostic traits of *G. kusukusense*, when compared with another dubious species *G. chademenosocarpum* also described in Taiwan, as suggested by Li (1994) based on the protologues of the two species. Thus the distribution of *G. kusukusense* in Taiwan was accepted by Hsu et al. (2006), although the type of the species was unavailable in their study. However, the detailed morphological description and line drawing of *G. kusukusense* provided in Hsu et al. (2006) are very different from the type of the species but well identical with *G. ellipticum*, a species distributed in Taiwan but omitted in most literature of Taiwanese *Glochidion*, including Hsu et al. (2006). The result from rechecking the specimens cited as *G. kusukusense* in Hsu et al. (2006) further confirmed our conclusion. More specimens of *G. ellipticum* collected from Chiayi, Kaosiung, Keelung, Pingtung, Nantou, Taichung and Tainan of Taiwan were also found and studied in the current study (see ‘Representative specimens examined’ under the species *G. ellipticum*).

Morphologically, the species *G. lanceolatum* is similar to the typical variety of *G. zeylanicum*, but differs by its smaller leaves (6–13 × 2.5–4 cm), ovaries 4–6-locular, and capsules 6–7 mm in diameter (Figure 2G). In contrast, G. zeylanicum var. zeylanicum has larger leaves (8.5–23.5 × 5–9 cm), ovaries 6–8-locular, and capsules 8–12 mm in diameter (Figure 2Q). Additionally, as revealed in previous studies, pollen morphology of the two taxa also showed differences in terms of pollen size and aperture system (Deng 1992; Yao and Zhang 2016). Pollen grains of *G. lanceolatum* are smaller in size [polar axis (P) = 17.25 μm, equatorial axis (E) = 15.47 μm] and showed a 3–4-colporate aperture pattern (Yao and Zhang 2016). In contrast, pollen grains of G. zeylanicum var. zeylanicum are larger in size (P = 22.28 μm, E = 19.49μm) and showed a 4-colporate aperture pattern (Yao and Zhang 2016).

#### Representative specimens examined.

China. Taiwan. Hsinchu Hsien, Peipu, 24°42'0"N, 121°3'5"E, 12 January 1908, *U. Mori s.n.* (TAI); Hualien Hsien, Tungmen, Wunlan, at an elevation of 180–250 m, 23 November 1982, *Y. Tateishi 16250* (HAST); Keelung, 25°7'43"N, 121°44'9"E, 8 September 1928, *S. Sasaki s.n.* (TAI); Kaohsiung, Shoushan, al. 400 m, 16 October 1985, *S.Y. Lu 17379* (HAST); Keelung, Ensorei, 25°7'32"N, 121°45'56"E, 14 April 1929, *S. Suzuki s.n.* (TAI); Keelung, Hopingtao, 25°9'33"N, 121°45'5"E, 8 December 1963, *C.C. Chuang & M.T. Kao 5541* (PH, TAI); Ilan Hsien, Lotung, 24°40'58"N, 121°47'13"E, 13 November 1932, *S. Suziki 12348* (TAI); Ilan Hsien, Suao, 24°35'34"N, 121°50'38"E, *Y.M. Hsu 113* (TAI); Ilan Hsien, Lungtanhu, along the paved road surrounding the lake, at an elevation of 100 m, 23 January 1997, *S.M. Liu 556* (HAST); Ilan Hsien, Toucheng Town, TaHSI, Taoyuanku trail, at an elevation of 50 m, 16 November 2000, *J.J. Chen 539* (HAST); Miaoli Hsien, Zhuolan, at an elevation of 0–300 m, 10 July 2001, *C.M. Wang 5060* (IBSC); Pingtung Hsien, Manchou Hsiang, Chunhsing Bridge-Chiatulu, 22°01'17"N, 120°48'29"E, at an elevation of 60–100, on broadleaf forest, 11 April 1998, *C.M. Wang et al. 03069* (PE); Pingtung Hsien, Peiyeh-Shanpaiwan, 22°42'6"N, 120°38'31"E, 25 December 1930, *S. Suzuki 6798* (TAI); Pingtung Hsien, Shihtzu Hsiang, Shouchia-Mutan, 22°14'46"N, 120°49'49"E, at an elevation of 420 m, roadside, 26 March 1999, *C.M. Wang 03936* (PE); Taipei, Muchihshan, 25°1'15"N, 121°35'3"E, 12 April 1985, *S.F. Huang 2780* (TAI); Taipei, Peitou, 25°7'42"N, 121°29'42"E, 13 December 1931, *T. Suzuki 5904* (TAI); Taipei, Tatungshan, 25°10'22"N, 121°31'33"E, 30 December 1929, *Y. Simada 1743C* (TAI); Taipei, Wantan, 24°56'39"N, 121°31'49"E, 21 March 1949, *H. Keng 1008* (TAI); Taipei, Chungho Shih: Yuan-Tung-Ssu, at an elevation of 50 m, 6 October 1989, *C.H. Lin 258* (HAST); Taipei, Linkou Hsien, Hou-hu, roadside, at an elevation of 100–200 m, 23 September 2000, *C.L. Huang & H.M. Chang 134* (HAST); Taitung Hsien, Hungtou river, Lanyu, 22°1'49"N, 121°33'13"E, *T. Hosokawa 8048* (TAI); Taitung Hsien, Lanyu Hsiang, Bridge Chungaichiao, roadside, 4 December 1996, *T.Y.A. Yang et al. 07749* (KUN); Taitung Hsien, Lanyu Hsiang, Langtao, Pond Hsiaotienchih, at an elevation of 180 m, roadside, 18 December 1997, *T.Y.A. Yang et al. 09881* (IBSC); Taitung Hsien, Lanyu Hsiang, Langtao, Pond Hsiaotienchih, at an elevation of 150–180 m, 9 July 1997, *T.Y.A. Yang et al. 08598* (IBSC); Taitung, Lanyu, Orchid Is., 22°3'23"N, 121°30'52"E, *T.C Huang et al. 10552* (TAI).

### 4. Glochidion lanyuense

Taxon classificationPlantaeMalpighialesPhyllanthaceae

Gang Yao & S.X. Luo sp. nov.

4C3E179F-595E-5403-B738-DD639DFE5B8D

urn:lsid:ipni.org:names:77211389-1

**Figure 4.**
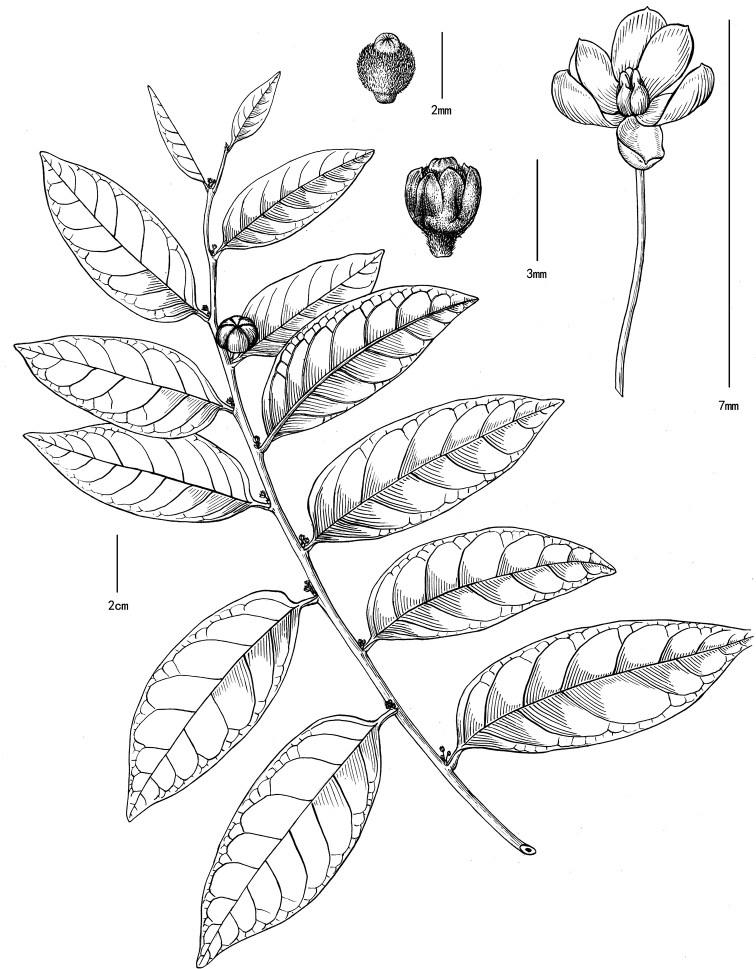
*Glochidion lanyuense* Gang Yao & S.X. Luo, sp. nov. (based on the holotype, drawn by Y.X. Liu) **A** habit **B** ovary and style **C** female flower **D** male flower.

**Figure 5.**
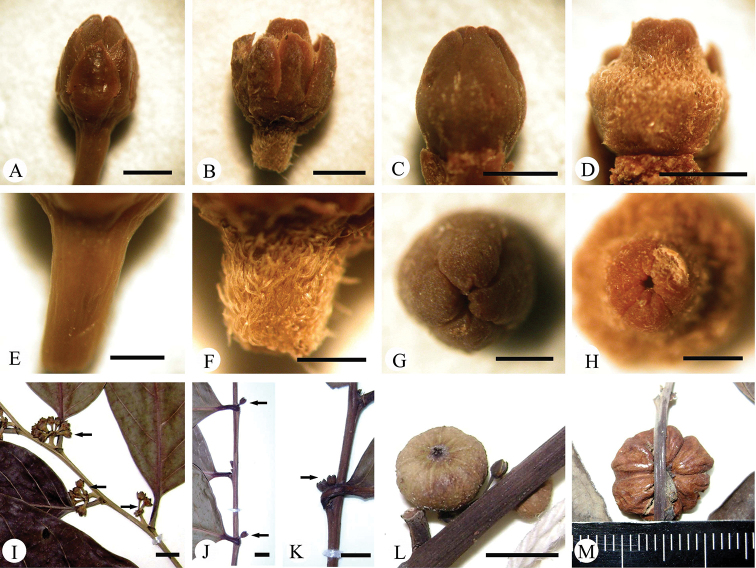
Morphological comparison between *Glochidion lanyuense* Gang Yao & S.X. Luo and *G. lanceolatum* Hayata **A, C, E, G, I, L***G. lanyuense***B, D, F, H, J, K, M***G. lanceolatum***A, B** female flower **C, D** ovary and style **E, F** pedicle of female flower **G, H** general view of style **I–K** female flowers (shown by arrowheads) **L, M** fruit. Scar bars: 1 mm (**A–H**); 3 mm (**I–K**); 5 mm (**L**).

Figures 4 , 5B, D, F, H, J, K, M

#### Diagnosis.

The species is morphologically similar to *G. lanceolatum*, but differs by its female flowers usually solitary or rarely two in axillary, pedicel of female flowers and ovaries usually densely strigose, styles ovoid column and strigose at base, and fruits ca. 10 mm in diameter.

#### Type.

China. Taiwan, Taitung Hien, Lanyu Hsiang, Hongtoucun, on roadsides of broadleaf forest, 22°01'00"N, 121°33'27"E, at an elevation of 1–10 m, 16 September 1998, *C.M. Wang 03521* (holotype: IBSC-0330741!).

#### Description.

Shrubs or treelets, monoecious; branchlets pubescent. Leaf blade oblong ovate, or elliptic, 6–10 × 3–4.5 cm, papery, slightly leathery, with apex acuminate or acute, and base broadly cuneate or rounded, glabrous in both surface; lateral veins 6–7 pairs, prominent beneath. Petiole 4–7 mm long, glabrous. Stipules broadly triangular, 1–1.5 mm long. Flowers in axillary solitarily or two. Male flowers: pedicles ca. 5 mm long, glabrous; sepals 6, oblong or ovate, biseriate, glabrous; stamens 3, 1–1.2 mm long. Female flowers: pedicles ca. 1 mm long, usually densely strigose; sepals 6, ovoid-triangular or ovate, biseriate, sparsely tomentose; ovary depressed globose, 5–6-locular, densely strigose; style connate into a cylindrical column, ca. 0.5 mm long, truncate at apex, densely strigose at base, 5–6-lobed apex, and then shallowly 2-lobed for each lobes. Capsules depressed globose, ca. 10 mm in diameter, sub-glabrous, 5–6-grooved.

#### Distribution and habitat.

The species is known only from its type locality, Lanyu island of Taiwan, China. It grows on roadsides of broadleaf forest at low altitude.

#### Etymology.

*Glochidion lanyuense* is named after its type locality, Lanyu island of Taiwan.

#### Taxonomic discussion.

*Glochidion lanyuense* is quite different from all of the other *Glochidion* species recorded from China, Japan, and the Philippines by its special characters of female flowers. It is similar to *G. lanceolatum* in habit, but differs (Table 1) in its female flowers which are usually solitary or rarely two in axillary (Figure 5J, K), pedicel of female flowers and ovaries are usually densely strigose (Figure 5B, D, F), style connate into a short cylindrical column (Figure 5D, H), stamens 3, capsules ca. 10 mm in diameter and 5–6-grooved (Figure 5M). In contrast, the species *G. lanceolatum* has multiple female flowers (usually 6–15) in supra-axillary cymes (Figures 2F, 5I) or rarely axillary, pedicel of female flowers and ovaries are glabrous (Figure 5A, C, E), styles sub-conical Figure 5C, G), stamens 4–6, fruits 6–7 mm in diameter and shallowly 4- or 6-grooved or obscurely grooved (Figures 2G, 5L). Additionally, as revealed in our previous palynological study (Yao and Zhang 2016), pollen grains of *G. lanyuense* (recorded as *Glochidion sp.3* in Yao and Zhang 2016) were much larger in size (P = 21.01 μm, E = 21.12 μm), 4-colporate in aperture system, and rugulate in exine ornamentation. While pollen grains of *G. lanceolatum* were smaller in size (P = 17.25 μm, E = 15.47 μm), 3–4-colporate, and regular reticulate in exine ornamentation, all of these characters are also consistent with the observation of Deng (1992). The rugulate ornamentation observed in pollen grains of the new species seems to be very different from those of other Taiwanese *Glochidion* species observed previously (Deng 1992; Yao and Zhang 2016).

**Table 1.** Morphological comparison between *Glochidion lanceolatum* Hayata and *G. lanyuense* Gang Yao & S.X. Luo.

Traits	*Glochidion lanceolatum*	*Glochidion lanyuense*
Female flower	6–15 female flowers usually in supra-axillary cymes or rarely axillary	Solitary or rarely two in axillary
Pedicel of female flower	Glabrous	Densely strigose
Ovary	Glabrous, or rarely pubescent	Densely strigose
Style	Sub-conical	Short cylindric column
Stamen	4–6	3
Fruit	Glabrous, 6–7 mm in diameter, usually grooved obscurely or shallowly 4–6-grooved	Sub-glabrous, ca. 10 mm in diameter, 5–6-grooved

### 5. Glochidion philippicum

Taxon classificationPlantaeMalpighialesPhyllanthaceae

(Cav.) C.B. Rob., in Philipp. J. Sci. Bot. 4: 103. 1909

C7766ADB-933A-5E27-96B5-4FC05BABA749

Figure 2I–K

Glochidion formosanum Hayata, in J. Coll. Sci. Univ. Tokyo 20: 20. tab. 2G. 1904. Type: China. Formosa (now Taiwan), Tai-tong-thian, Pi-Iam, 23 December 1899, *K. Miyake s.n.*, (holotype: TI-01802, photo!).
Bradleia philippica Cav., in Icon. 3: 48. tab. 371. 1797. Basionym.

#### Type.

Philippines, *L. Née s.n.* (lectotype: MA-475455, photo!, designated by Yao et al. 2017; isolectotype: MA-475454, photo!).

#### Distribution and habitat.

*Glochidion philippicum* is distributed in China (only in Taiwan), Malaysia, and the Philippines. The species usually occurs beside a gravelly road, forest margins, and roadsides, at low and medium altitudes. In Taiwan, the species is widely distributed from Ilan, Taichung, Changhua and Chiayi, to Hualian, Kaohsiung, Pingtung, Tainan and Taitung.

#### Taxonomic discussion.

*Glochidion philippicum* differs from all other *Glochidion* species in Taiwan by its sub-hemispherical or sub-conical styles (Figure 2I), capsules that are deeply 10–16-grooved, and persistent sub-hemispheric styles (Figure 2K).

#### Representative specimens examined.

China. Taiwan. Changhua, 30 November 1991, *M.J. Deng 751* (HAST); Changhua, Pakuashan, at an elevation of 50–200 m, 7 April 1991, *S.L. Chen 382* (HAST); Chiayi, Chungpu, 23°25'6"N, 120°30'57"E, December 1934, *K. Mori 2339* (TAI); Chiayi, Chuchi District, Kuanghua Village: Chiehtung, at an elevation of 600–800 m, 26 October 1985, *C.I. Peng 8762* (HAST); Hualian Hsien, Chuolu, at an elevation of 250 m, 17 November 1982, *Y. Tateishi & J. Murata 15540* (IBSC); Hualien, Sanmin, 23°26'51"N, 121°24'25"E, 15 December 1939, *Suzuki-Tokio 19781* (TAI); Hualien Hsien, Wanjung District, Hungyeh Village, Hungyeh Hot Spring, at an elevation of ca. 200–400 m, 3 July 1988, *C.I. Peng et al. 11618* (PE); Hualian Hsien, Zuepei, at an elevation of 250 m, 18 November 1982, *Y. Tateishi & J. Murata 15583* (IBSC); Ilan Hsien, Lotung, at an elevation of 10–20 m, 10 February 1992, *S.L. Chen 807* (HAST); Kaohsiung, 22°37'39"N, 120°16'55"E, 7 August 1938, *Tsuchiya 27* (TAI); Kaohsiung, Chaishan, broadleaf forest on mountain slope, at an elevation of 100 m, 24 June 1999, *K.F. Chung 1389* (HAST); Pingtung Hsien, Hengchun Town, Kengting Park, 21°58'12"N, 120°48'27"E, at an elevation of 300 m, 15 July 1997, *C.M. Wang & H.M. Lin 02675* (IBSC, PE); Pingtung Hsien, Kentin, at an elevation of 300 m, 16 August 1969, *Y. Ando et al. 601* (KUN); Pingtung, Kenting, 21°57'6"N, 120°47'26"E, 26 September 1966, *C.C. Chuang & M.T. Kao 3946* (TAI); Pingtung, Oluanpi, South Cape, 21°54'9"N, 120°50'45"E, 30 December 1928, *Y. Kudo & S. Suzuki 15811* (TAI); Pingtung Hsien, Mt. Nanjen-shan, at an elevation of 450 m, 3 November 1982, *H. Ohashi & Y. Tateishi 13495* (IBSC); Pingtung Hsien, Mutan Hsiang, Kaoshih-Kaoshihfo, 22°07'39"N, 120°49'35"E, at an elevation of 200–300 m, beside a gravelly road, 5 September 1998, *C.M. Wang 03487* (IBSC, PE); Pingtung, Sheting Nature Park, 21°57'20"N, 120°48'32"E, 22 November 1984, *J.C. Wang 2656* (TAI); Pingtung Hsien, Wutai Hsiang, on the way from Haocha to Old Haocha, 22°42'37"N, 120°41'31"E, at an elevation of 250–430 m, roadside, 19 July 1995, *T.Y. Liu et al. 771* (IBSC, PE); Taichung, Fungyuan, secondary forest, roadside, at an elevation of 300–350 m, 18 July 1991, *M.J. Deng 609* (HAST); Tainan, Chentoushan, 23°19'50"N, 120°30'2"E, 20 June 1937, *Mori 2329* (TAI); Tainan, Mado, 23°10'38"N, 120°13'36"E, 10 August 1988, *S.F. Huang & T.C. Huang 13740* (TAI); Tainan, Nanhsi Hsiang, along a paved road to Hsienkungmiao, at an elevation of 300–500 m, 16 October 2002, *P.J. Lin 74* (HAST); Taitung Hsien, Chihen Hot Spring, 22°41'46"N, 120°59'49"E, 1967, *C.C. Hsu & M.T. Kao 3382* (TAI); Taitung, Kannatolo, 22°51'38"N, 121°7'0"E, 28 July 1937, *Y. Yamamoto & K. Mori s.n.* (TAI).

### 6. Glochidion puber

Taxon classificationPlantaeMalpighialesPhyllanthaceae

(L.) Hutch., in Sarg. Pl. Wilson. 2: 518. 1916 [as G. puberum]

F0B22E28-0582-5ABA-9407-0EA55BEAFF00

Figure 2L, P

Agyneia pubera L., Mant. 2: 296. 1771. Basionym.

#### Type.

China. *Anon s.n.* (holotype: LINN, sheet no. LINN-1145.2, photo!).

#### Distribution and habitat.

*Glochidion puber* is widely distributed in China and also recorded in Kyushu of Japan. It occurs usually on slopes, or in scrub on stream banks, forest margins, roadsides, at altitudes between 100 and 2200 m. In Taiwan, the species is distributed from Miaoli, to Changhua, Nantou and Taichung.

#### Taxonomic discussion.

The species differs from all other *Glochidion* species in Taiwan by its annular styles (Figure 2L), and the persistent annular styles on capsules which are not, or only slightly, elevated (Figure 2P).

#### Representative specimens examined.

China. Taiwan. Detailed locality unknown, 23 October 1929, *Anonymous s.n.* (PE-00961458); Detailed locality unknown, 24 October 1929, *Anonymous s.n.* (IBSC-0314244); Changhua Hsien, Puhsin, 13 October 1988, *S.M. Chaw 742* (HAST); Miaoli Hsien, Cholan Town, the First Cemetery, at an elevation of 450 m, 1 November 2008, *P.F. Lu 17251* (HAST); Nantou Hsien, Chungming, 23°52'50"N, 120°54'42"E, 23 September 1929, *K. Sasaki 15713* (TAI); Nantou Hsien, Sun Moon Lake, 23°50'26"N, 120°55'26"E, 20 September 1929, *K. Sasaki 15509* (TAI); Nantou Hsien, Yuechih-Sunmoonlake, 23°52'35"N, 120°55'5"E, 23 October 1930, *S. Suzuki 6513* (TAI); Nantou Hsien, 8 June 1991, *Y.S. Hsu* & *J.C. Liaw 222* (NCAI); Nantou Hsien, 24 August 1991, *Y.S. Hsu* & *J.C. Liaw 250*, *251*, *252*, 253, *255*, *256*, *258 & 259* (NCAI); Nantou Hsien, Yuchi Hsiang, Sun-moon-lake, at an elevation of 700 m, 2 November 2007, *P.F. Lu 14821* (HAST); Taichung Hsien, 27 April 1991, *Y.S. Hsu* & *J.C. Liaw 185* (NCAI); Taichung Hsien, 2 November 1933, *Suzuki-Tokio 10796* (NAS); Taichung, Shihpikeng, 24°18'6"N, 120°46'26"E, 15 December 1922, *S. Suzuki s.n.* (TAI); Taichung, Fengyuan, secondary forest, roadside, at an elevation of 350–450 m, 18 July 1991, *M.J. Deng 605* (HAST).

### 7. Glochidion rubrum

Taxon classificationPlantaeMalpighialesPhyllanthaceae

Blume in Bijdr. Fl. Nederl. Ind. 586. 1825

9714DB4A-8D73-5652-A9BE-2A823709AF64

Figures 1D–F , 2M–O

Glochidion chademenosocarpum Hayata in Icon. Pl. Formos. 9: 94. 1920. syn. nov. Type: China. Formosa (now Taiwan), Inter Onô et Kôsenpo, October 1917, *B. Hayata s.n.* (holotype: TI-01801, photo!, Figure 1D).
Glochidion fortunei Hance var. longistylum H. Keng in Journ. Acard. Washington Sci. 41(6): 200. 1951. Type: China. Taiwan, Kaohsiung, 14 August 1937, *Yamomoto & Mori 790* (holotype: TAI, photo!).
Glochidion fortunei Hance var. megacarpum H. Keng in Journ Acad. Washington Sci. 41(6): 200. 1951. Type: China. Taiwan, Kaoshiung, 8 April 1929, *Kudo* & *Suzuki 96* (holotype: TAI).
Glochidion suishaense Hayata in Icon. Pl. Formos. 9: 97. 1920. Type: China. Formosa (now Taiwan), Suisha, 29 Apr. 1916, *B. Hayata s.n.* (lectotype: TI-01820, photo!, here designated; isolectotype: TI-01821, photo! Figure 1F); Remaining syntype: Taiwan, *B. Hayata s.n.* (TI-01823 & TI-01824, photos!).

#### Type.

Indonesia, Java, *C.L. von Blume s.n.* (holotype: not traced; isotypes: CAL; NY-00263451, photo!, Figure 1E).

#### Distribution and habitat.

*Glochidion rubrum* is recorded widely from India to Cambodia, China, south Japan, Malaysia, Indonesia, Philippines and Vietnam. In China, it occurs in Anhui, Fujian, Guangdong, Hainan, Hong Kong, Taiwan and Zhenjiang. It grows in broad-leaved evergreen forests, roadsides from low altitude to 1800 m. The species is widely distributed from northern to southern Taiwan.

#### Taxonomic discussion.

Hayata (1920) described *G. chademenosocarpum* based on one of his collections (*B. Hayata s.n.*, TI, photo!; Figure 1D) from Taiwan, and he suggested that the species differed from *G. rubrum* (recorded as *G. fortunei* in his study) by its much more densely clustered and sessile female flowers. After checking the protologue and observing the type of *G. chademenosocarpum* (Figure 1D), it was concluded that the species is well conspecific with *G. rubrum* (Figure 1E) and should be treated as a new synonym of the latter. Based on morphological description (Hayata 1920) as well as our observation of the type, we found that the type of *G. chademenosocarpum* might represent an unripe flower branch of *G. rubrum* because the morphology of branch, leaves, ovaries and styles observed are all identical with those of *G. rubrum*, except the ovaries and styles are smaller in size compared with those of the ripe female flowers described by Hsu et al. (2006) as well as those observed in living plants. Additionally, most male flowers observed from the type of *G. chademenosocarpum* also seem to be unripe because sepals of most male flowers were unopen (Figure 1D).

The taxonomic history of *G. suishaense* was similar to that of *G. chademenosocarpum* and *G. kusukusense*, except Hsu et al. (2006) treated it as a synonym of *G. rubrum* based on its protologue. In the present study, the result from observing the type of *G. suishaense* (Figure 1F) further confirmed its taxonomic status.

Morphologically, *G. rubrum* could be distinguished from all other Taiwanese *Glochidion* species by its styles which are cylindrical in shape and 1–3 mm in length (Figure 2M).

#### Representative specimens examined.

China. Taiwan. Chiayi, at an elevation of 700–1300 m, 1 November 1985, *C.I. Peng 8789* (HAST); Hsinchu, Senkyakuseki, 25 June 1927, Y. Simada 4147 (HAST); Hsinchu, Lienhuassu, at an elevation of 50–100 m, 30 August 1996, *K.C. Yang 4908* (HAST); Hualien Hsien, Yueh-wang-ting to Yen-hai logging tract, 3 April 1991, *J.C. Wang et al. 6757* (HAST); Hualien Hsien, Hsiulin Hsiang, Hoping Forest Road, 24°18'26"N, 121°41'57"E, at an elevation of ca. 875 m, 22 August 1996, *S.M. Liu et al. 362* (PE); Ilan Hsien, Mohen, 24°26'54"N, 121°37'30"E, 30 September 1930, *S. Suzuki 6097* (TAI); Ilan Hsien, Nanao Hsiang, on the way from hiking entrance to Machialanshan, at an elevation of 270 m, 13 January 1994, *Y.R. Lin 294* (HAST); Ilan Hsien, Nanaonanhsi, at an elevation of 440 m, 16 August 1995, *T.Y. Liu 817* (HAST); Kaosiung Hsien, Taoyuan Hsiang, southern Cross-Island Hwy near Likuan, broadleaf forest, 23°16'57"N, 120°52'24"E, at an elevation of ca. 1800 m, 1 April 1995, *T.Y. Liu et al. 509* (IBSC); Nantou Hsien, Chen-you-lan-chi, at an elevation of 1650 m, 7 May 1988, *W.H. Hu 663* (IBSC); Pingtung, Kaoshih, 22°7'54"N, 120°50'42"E, 1 January 1929, *S. Suzuki 16046* (TAI); Pingtung Hsien, Mutan Hsiang, Gaushr-Mutan, 22°08'22"N, 120°49'49"E, at an elevation of 250 m, roadside, 26 March 1999, *C.M. Wang 03970* (IBSC, PE); Pingtung Hsien, Mutan Hsiang, Mutan-Hsushai, 22°11'13"N, 120°51'17"E, on roadside, 12 April 1998, *C.M. Wang et al. 03099* (IBSC); Pingtung Hsian, Shihtzu Hsiang, on the way from Neiwen to Shouka, along Hsien road 199, broadleaf forest, 22°13'57"N, 120°51'58"E, at an elevation of ca. 390 m, 6 December 1995, *S.M. Liu et al. 140* (PE); Pingtung Hsien, Tahanshan, 22°24'N, 120°46'E, at an elevation of 600 m, 20 September 1996, *T.T. Chen 7883* (PE); Taichung Hsien, Hoping Hsiang, on Hsuehshan forest, at road mileage sign 23.7 km, 24°14'57"N, 120°55'30"E, at an elevation of 1600 m, 5 May 1999, *S.H. Wu 1277* (KUN); Taichung Hsien, Hoping Hsiang, at an elevation of ca. 1100 m, 29 May 1999, *C.H. Chen et al. 2737* (HAST); Tainan, Lungtien, 23°12'5"N, 120°16'33"E, 17 May 1942, *Senbenlin 303* (TAI); Taipei Hsien, Nankang, local hills up the Hu-Shih Park, at an elevation of ca. 50–100 m, 14 April 1991, *C.I. Peng et al. 13898* (PE); Taipei, Peitou, 25°7'42"N, 121°29'42"E, 5 May 1935, *H. Shimada 360* (TAI); Taipei Hsien, Yangmingshan National Park, Tatunshan, 25°22'00"N, 121°31'31"E, at an elevation of ca. 825–840 m, on exposed trail, 10 November 1994, *H.Y. Shen et al. 275* (KUN); Taitung Hsien, Lanyu Hsiang, Hsiangtienchih, 22°04'50"N, 121°30'05"E, at an elevation of 180 m, semi-shaded, 28 April 1997, *S.T. Chiu & J.N. Chen 04099* (IBSC); Taitung Hsien, Lanyu Hsiang, Langtao, Pond Hsiaotienchih, at an elevation of 150–180 m, 9 July 1997, *T.Y.A. Yang et al. 08597* (IBSC); Taitung Hsien, Lutao Hsiang, along the paved road from Nanliao to Huoshaoshan, at an elevation of 100 m, 9 October 2001, *Y.Y. Huang 753* (PE); Taoyuan Hsien, Fuhsiang Hsiang, Litungshanchuang-Shankuang, 24°40'47"N, 121°20'23"E, at an elevation of 960 m, on roadside of broadleaf forest, 4 January 1996, *C.M. Wang & H.M. Lin 01953* (IBSC); Taoyuan Hsien, Nankan, 24°59'17"N, 121°18'22"E, 5 May 1929, *Y. Yamamoto s.n.* (TAI).

### 8. Glochidion zeylanicum

Taxon classificationPlantaeMalpighialesPhyllanthaceae

(Gaertn.) A. Juss., in Tent. Euphor. 107. 1824

024AEC2B-C7B5-50E9-8A9A-BFC0F593F0C8

Bradleia zeylanica Gaertn., in Fruct. 2: 128. 1791.Basionym.

#### Type.

Gaertner, Fruct. Sem. Pl. 2: t. 109.1791 (lectotype designated by Chakrabarty and Gangopadhyay 1995).

### a. var. zeylanicum

Taxon classificationPlantaeMalpighialesPhyllanthaceae

4C37D5A4-E129-5E66-93EF-BFEB54AAE7DF

Figure 2Q–S

#### Distribution and habitat.

The typical variety G. zeylanicum var. zeylanicum is widely distributed from India, Sri Lanka, through Myanmar, Thailand, Vietnam, to China, south Japan, Indonesia, and the Pacific islands. In China, it occurs widely from the southwest of the mainland to Taiwan island. It usually grows in sparse forests, margins of woods, humid valleys, scrub on stream banks, roadsides, and at low and medium altitudes. In Taiwan, the variety is widely distributed from Hsinchu, Ilan, Taoyuan and Taipei, to Nantou, Taichung and Pingtung.

#### Taxonomic discussion.

The typical variety G. zeylanicum var. zeylanicum is similar to *G. lanceolatum* in habit, and morphological differences between them have been discussed under the latter species.

#### Representative specimens examined.

China. Taiwan. Hsinchu Hsien, Kuanhsi, at an elevation of 200 m, 24 September 1985, *S.Y. Lu 17142* (HAST); ILan Hsien, Chiaohsi Hsiang, Lungtanhu, 24°48'00"N, 121°44'06"E, at an elevation of ca. 100 m, slope above the road with some trees, 23 January 1997, *S.M. Liu et al. 556* (PE); ILan Hsien, Yuanshan, roadside, 15 April 1991, *M.J. Deng 404* (HAST); Nantou, Meifeng Farm, 24°6'0"N, 121°10'55"E, 1 August 1939, *Masamune et al. 2441* (TAI); Nantou, Sun Moon Lake, 23°50'26"N, 120°55'26"E, 24 October 1930, *S. Suzuki 6722* (TAI); Pingtung, Kengting, at an elevation of 200–300 m, *M.J. Deng & S.L. Chen 834* (HAST); Taichung Hsien, Wufeng Hsiang, Tingtai, 24°03'15"N, 120°40'24"E, open place, 22 January 2000, *J.N. Chen 00047* (PE); Taihoku, September 1922, *S. Sasaki s.n.* (NAS); Taipei, Chihshanyen, 25°5'38"N, 121°30'57"E, 15 May 1932, *T. Nonaka & K. Mori s.n.* (TAI); Taipei, NTU campus, 25°0'57"N, 121°32'9"E, 9 February 1964, *J.G. Kung 42* (TAI); Taipei, Tanshui, 25°9'50"N, 121°26'11"E, 10 December 1921, *S. Sasaki 1910* (TAI); Taipei, Taihoku, 25°2'46"N, 121°30'43"E, September 1922, *S. Sasaki 1911* (TAI); Taipei, Taihoku, 25°2'46"N, 121°30'43"E, 2 August 1927, *Y. Shimada 3404* (TAI); Taipei, Wantan, 24°56'39"N, 121°31'49"E, 18 June 1936, *H. Siizu 2342* (TAI); Taipei, Nei-Shuang-His, 12 December 1997, *M.F. Kao 3304* (HAST); Taoyuan Hsien, Lungtan, at an elevation of 220 m, 21 July 1990, *C.H. Lin 13301* (HAST, PE); Taoyuan Hsien, Yangmei, roadside, at an elevation of 50–150 m, 12 December 1990, *M.J. Deng 25* (HAST); Taoyuan Hsien, Gueishan, Fongshu, at an elevation of 100–200 m, 28 September 2002, *C.C. Chen 458* (HAST).

### b. var. tomentosum

Taxon classificationPlantaeMalpighialesPhyllanthaceae

Trim., in Cat. Ceyl. Pl. 79. 1885

04FA54DB-780F-5B58-B5BF-116618B00618

Figure 2T–V

#### Type.

Sri Lanka, *G.H.K. Thwaites 3432* (lectotype: BM-000617461, designated by Chakrabarty and Balakrishnan 2018; Isolectotypes: CAL, K-001081200 & K001081201).

#### Distribution and habitat.

This variety G. zeylanicum var. tomentosum is widely distributed from India, Myanmar, Thailand, Vietnam, to China and south Japan. In China, it occurs widely from southwestern areas to Taiwan. It shares a similar habitat with the typical variety G. zeylanicum var. zeylanicum. In Taiwan, G. zeylanicum var. tomentosum is distributed from Hsinchu and Taipei to Nantou. Hsu et al. (2006) recorded the distribution of this variety in Ilan Hsien, but relevant specimens were unavailable in the present study.

#### Taxonomic discussion.

Morphologically, the variety G. zeylanicum var. tomentosum differs from the typical variety G. zeylanicum var. zeylanicum by its hairy habit. For the hairy taxon, the name G. zeylanicum var. tomentosum was accepted by several authors in their treatment of Taiwanese *Glochidion* (Hsieh 1977; Deng and Wang 1993) and further appreciated recently (TPL 2013 continuously updated, Yao and Zhang 2015a), but in some other treatments the name *G. hirsuttum* (Roxb.) Voigt was adopted (Li 1994; Hsu et al. 2006; Li and Gilbert 2008). In our taxonomic study of the genus *Glochidion*, it was found that the hairy variety (Figure 2T–V) and the typical glabrous variety G. zeylanicum var. zeylanicum (Figure 2Q–S) are very similar in habit and also shared similar distribution areas, so the name G. zeylanicum var. tomentosum is accepted here.

#### Representative specimens examined.

China. Taiwan. Detailed locality unknown, 10 June 1929, *S. Sasaki s.n.* (NAS); Hsinchu Hsien, Hsinfeng Hsiang, Fengshan Margin of fallow paddy, at an elevation of 5–10 m, 30 May 1991, *W.P. Leu 946* (HAST); Hsinchu Hsien, Chupei Hsiang, on the slope along the riverbank of Fengshanhsi, at an elevation of 50–100 m, 27 November 1992, *W.P. Leu 1645* (HAST); Hsinchu Hsien, Hsinfeng Hsiang, 22 March 2014, *P.M. Zeng PM14* (NCAI); Nantou Hsien, Hsianshan-Sunmoon Lake, 23°50'29"N, 120°53'19"E, 19 September 1929, *K. Sasaki 15394* (TAI); Nantou Hsien, Sun Moon Lake, 23°50'26"N, 120°55'26"E, September 1929, *S. Sasaki s.n.* (TAI); Nantou Hsien, Yuchih, at an elevation of 750 m, 25 December 1985, *S.Y. Lu 18170* (HAST); Nantou Hsien, 26 April 1991, *Y.S. Hsu* & *J.C. Liaw 174* (NCAI); Nantou Hsien, 8 June 1991, *Y.S. Hsu* & *J.C. Liaw 216* (NCAI); Taipei Hsien, Shuiyuanti, 25°0'27"N, 121°31'48"E, 14 May 1929, *S. Suzuki 19294* (TAI); Taipei Hsien, Sungshan, 25°2'53"N, 121°34'5"E, 27 April 1933, *S. Sasaki s.n.* (TAI); Taipei Hsien, Taihoku, 25°2'46"N, 121°30'43"E, 10 June 1929, *S. Sasaki 9294* (TAI); Taipei Hsien, Tomitacho, 25°0'43"N, 121°32'7"E, 27 May 1932, *T. Tanaka & Y. Shimada 11071* (TAI); Tomita-cho, Taihoku-shi, 27 May 1932, *T. Tanaka et al. 11071* (IBSC, PE); Taipei Hsien, Kungkuan, roadside, at an elevation of 10–20 m, 20 March 1992, *M.J. Deng 883* (HAST).

### Key to species of *Glochidion* in Taiwan, China

**Table:** 

1	Female flowers in axillary clusters; stamens usually 3	**2**
–	Female flowers usually supra-axillary cymes or rarely in axillary clusters; stamens more than 3	**7**
2	Ovary usually 3–4-locular, or rarely 5-locular	**3**
–	Ovary 5-locular or more than 5-locular	**4**
3	Leaves glaucous and white pubescent abaxially; styles column cylindric; capsules deeply 6–8-grooved; persistent styles obvious, ca. 1 mm long, dilated at apex	**G. acuminatum var. acuminatum Müll. Arg.**
–	Leaves usually paler abaxially; styles column shortly conical; capsules grooved shallowly or obscurely; persistent styles obscure or slightly elevated	***G. ellipticum* Wight**
4	Styles cylindrical, 1–3 mm long; capsules glabrous, 6–10 mm in diameter	***G. rubrum* Blume**
–	Styles not cylindrical, usually less than 1 mm long; capsules hairy or slightly pubescent, up to 10 mm in diameter	**5**
5	Female flower usually solitary or rarely two in axillary; styles ovoid column; ovary 5–6-locular	***G. lanyuense* Gang Yao & S.X. Luo**
–	Female flowers multiple (usually more than 5) in axillary; styles annular, caliciform or sub-conical; ovary more than 6-locular	**6**
6	Lateral veins of leaves 6–7 pairs; styles annular; ovary 6–10-locular; capsules 6–10-grooved, usually reddish when mature; persistent styles annular, not or slightly elevated	***G. puber* (L.) Hutch.**
–	Lateral veins of leaves 8–9 pairs; styles caliciform or sub-conical; ovary 5–8-locular; capsules 10–16-grooved, usually purplish when mature; persistent styles sub-conical or sub-hemispheric	***G. philippicum* (Cavan.) C.B. Rob.**
7	Leaves less than 15 cm long and 5 cm wide; capsules 6–7 mm in diameter	***G. lanceolatum* Hayata**
–	Leaves up to 20 cm long and 8 cm wide; capsules 8–12 mm in diameter	**8**
8	Plant glabrous (except ovary)	**G. zeylanicum var. zeylanicum (Gaertn.) A. Juss.**
–	Plant hairy	**G. zeylanicum (Gaertn.) A. Juss. var. tomentosum Trim.**

## Supplementary Material

XML Treatment for Glochidion acuminatum

XML Treatment for var. acuminatum

XML Treatment for Glochidion ellipticum

XML Treatment for Glochidion lanceolatum

XML Treatment for Glochidion lanyuense

XML Treatment for Glochidion philippicum

XML Treatment for Glochidion puber

XML Treatment for Glochidion rubrum

XML Treatment for Glochidion zeylanicum

XML Treatment for var. zeylanicum

XML Treatment for var. tomentosum
